# Predicting Diabetes Mellitus With Machine Learning Techniques

**DOI:** 10.3389/fgene.2018.00515

**Published:** 2018-11-06

**Authors:** Quan Zou, Kaiyang Qu, Yamei Luo, Dehui Yin, Ying Ju, Hua Tang

**Affiliations:** ^1^School of Computer Science and Technology, Tianjin University, Tianjin, China; ^2^Institute of Fundamental and Frontier Sciences, University of Electronic Science and Technology of China, Chengdu, China; ^3^School of Medical Information and Engineering, Southwest Medical University, Luzhou, China; ^4^School of Information Science and Technology, Xiamen University, Xiamen, China; ^5^Department of Pathophysiology, School of Basic Medicine, Southwest Medical University, Luzhou, China

**Keywords:** diabetes mellitus, random forest, decision tree, neural network, machine learning, feature ranking

## Abstract

Diabetes mellitus is a chronic disease characterized by hyperglycemia. It may cause many complications. According to the growing morbidity in recent years, in 2040, the world’s diabetic patients will reach 642 million, which means that one of the ten adults in the future is suffering from diabetes. There is no doubt that this alarming figure needs great attention. With the rapid development of machine learning, machine learning has been applied to many aspects of medical health. In this study, we used decision tree, random forest and neural network to predict diabetes mellitus. The dataset is the hospital physical examination data in Luzhou, China. It contains 14 attributes. In this study, five-fold cross validation was used to examine the models. In order to verity the universal applicability of the methods, we chose some methods that have the better performance to conduct independent test experiments. We randomly selected 68994 healthy people and diabetic patients’ data, respectively as training set. Due to the data unbalance, we randomly extracted 5 times data. And the result is the average of these five experiments. In this study, we used principal component analysis (PCA) and minimum redundancy maximum relevance (mRMR) to reduce the dimensionality. The results showed that prediction with random forest could reach the highest accuracy (ACC = 0.8084) when all the attributes were used.

## Introduction

Diabetes is a common chronic disease and poses a great threat to human health. The characteristic of diabetes is that the blood glucose is higher than the normal level, which is caused by defective insulin secretion or its impaired biological effects, or both (Lonappan et al., 2007). Diabetes can lead to chronic damage and dysfunction of various tissues, especially eyes, kidneys, heart, blood vessels and nerves (Krasteva et al., 2011). Diabetes can be divided into two categories, type 1 diabetes (T1D) and type 2 diabetes (T2D). Patients with type 1 diabetes are normally younger, mostly less than 30 years old. The typical clinical symptoms are increased thirst and frequent urination, high blood glucose levels (Iancu et al., 2008). This type of diabetes cannot be cured effectively with oral medications alone and the patients are required insulin therapy. Type 2 diabetes occurs more commonly in middle-aged and elderly people, which is often associated with the occurrence of obesity, hypertension, dyslipidemia, arteriosclerosis, and other diseases (Robertson et al., 2011).

With the development of living standards, diabetes is increasingly common in people’s daily life. Therefore, how to quickly and accurately diagnose and analyze diabetes is a topic worthy studying. In medicine, the diagnosis of diabetes is according to fasting blood glucose, glucose tolerance, and random blood glucose levels (Iancu et al., 2008; Cox and Edelman, 2009; American Diabetes Association, 2012). The earlier diagnosis is obtained, the much easier we can control it. Machine learning can help people make a preliminary judgment about diabetes mellitus according to their daily physical examination data, and it can serve as a reference for doctors (Lee and Kim, 2016; Alghamdi et al., 2017; Kavakiotis et al., 2017). For machine learning method, how to select the valid features and the correct classifier are the most important problems.

Recently, numerous algorithms are used to predict diabetes, including the traditional machine learning method (Kavakiotis et al., 2017), such as support vector machine (SVM), decision tree (DT), logistic regression and so on. Polat and Günes (2007) distinguished diabetes from normal people by using principal component analysis (PCA) and neuro fuzzy inference. Yue et al. (2008) used quantum particle swarm optimization (QPSO) algorithm and weighted least squares support vector machine (WLS-SVM) to predict type 2 diabetes Duygu and Esin (2011) proposed a system to predict diabetes, called LDA-MWSVM. In this system, the authors used Linear Discriminant Analysis (LDA) to reduce the dimensions and extract the features. In order to deal with the high dimensional datasets, Razavian et al. (2015) built prediction models based on logistic regression for different onsets of type 2 diabetes prediction. Georga et al. (2013) focused on the glucose, and used support vector regression (SVR) to predict diabetes, which is as a multivariate regression problem. Moreover, more and more studies used ensemble methods to improve the accuracy (Kavakiotis et al., 2017). Ozcift and Gulten (2011) proposed a newly ensemble approach, namely rotation forest, which combines 30 machine learning methods. Han et al. (2015) proposed a machine learning method, which changed the SVM prediction rules.

Machine learning methods are widely used in predicting diabetes, and they get preferable results. Decision tree is one of popular machine learning methods in medical field, which has grateful classification power. Random forest generates many decision trees. Neural network is a recently popular machine learning method, which has a better performance in many aspects. So in this study, we used decision tree, random forest (RF) and neural network to predict the diabetes.

## Materials and Methods

### Data

The dataset was obtained from hospital physical examination data in Luzhou, China. This dataset is divided two parts: the healthy people and the diabetes. There are two healthy people physical examination data. We used one of healthy people physical examination data that contains 164431 instances as the training set. In the other data set, 13700 samples were randomly selected as an independent test set. The physical data include 14 physical examination indexes: age, pulse rate, breathe, left systolic pressure (LSP), right systolic pressure (RSP), left diastolic pressure (LDP), right diastolic pressure (RDP), height, weight, physique index, fasting glucose, waistline, low density lipoprotein (LDL), and high density lipoprotein (HDL). In the training dataset, there are many missing data. We deleted the abnormal and missing samples to reduce the impact of data processing on result. Consequently, we got 151598 diabetic physical data and 69082 healthy people physical data. So, we randomly selected 68994 healthy people and diabetic patients’ data, respectively as training set. Due to the data unbalance, we randomly extracted 5 times. The final result was the mean value of 5 experiments. The 13,700 patients physical examination data, which were randomly selected as the independent test set, were different from the previous five sets which were used as training set.

Another dataset is Pima Indians diabetics data (Jegan, 2014). In particular, all patients are females at least 21 years old of Pima Indian heritage. The dataset contains 8 attributes which are times of pregnancy, plasma glucose concentration after an 2-h oral glucose tolerance test, diastolic blood pressure, triceps skin fold thickness, 2-h serum insulin, body mass index, diabetes pedigree function and age. In this dataset, the original 786 diabetics data reduces to 392 after deleted the missing data.

### Classification

In this section, we used decision tree, RF and neural network as the classifiers. Decision tree and RF can implement in WEKA, which is a free, non-commercial, open source machine learning and data mining software based on JAVA environment. Neural network can be implemented in MATLAB, which is a commercial mathematics software exploited by MathWorks, Inc. It is used for algorithmic development, data visualization, data analysis and provides advanced computational language, and interactive environment for numerical calculation.

#### Decision Tree

Decision tree is a basic classification and regression method. Decision tree model has a tree structure, which can describe the process of classification instances based on features (Quinlan, 1986). It can be considered as a set of if-then rules, which also can be thought of as conditional probability distributions defined in feature space and class space.

Decision tree uses tree structure and the tree begins with a single node representing the training samples (Friedl and Brodley, 1997; Habibi et al., 2015; Liao et al., 2018). If the samples are all in the same class, the node becomes the leaf and the class marks it. Otherwise, the algorithm chooses the discriminatory attribute as the current node of the decision tree. According to the value of the current decision node attribute, the training samples are divided into serval subsets, each of which forms a branch, and there are serval values that form serval branches (Quinlan, 1986; Kohabi, 1996). For each subset or branch obtained in the previous step, the previous steps are repeated, recursively forming a decision tree on each of the partitioned samples (Quinlan, 1986; Friedl and Brodley, 1997; Habibi et al., 2015).

The typical algorithms of decision tree are ID3, C4.5, CART and so on. In this study, we used the J48 decision tree in WEKA. J48 another name is C4.8, which is an upgrade of C4.5. J48 (Salzberg, 1994; Kohabi, 1996) is a top-down, recursive divide and conquer strategy. This method selects an attribute to be root node, generates a branch for each possible attribute value, divides the instance into multiple subsets, and each subset corresponds to a branch of the root node, and then repeats the process recursively on each branch (Kohabi, 1996). When all instances have the same classification, the algorithm stop. In J48, the nodes are decided by information gain. According to the following formulas, in each iteration, J48 calculates the information gain of each attribute, and selects the attribute with the largest value of information gain as the node of this iteration (Quinlan, 1996a,b; Sharma et al., 2014).

Attribute *A* information gain:

$$Gain(A)=Info(D)-{Info}_{A}\left(D\right)$$

Pre-segmentation information entropy:

$$Info(D)=Entropy(D)=-\underset{j}{\Sigma }p(j|D)\log p(j|d)$$

Distributed information entropy:

$${Info}_{A}(D)=\overset{v}{\underset{i=1}{\Sigma }}\frac{n_{i}}{n}Info(D_{i})$$

#### Random Forest

RF is a classification by using many decision trees. This algorithm proposed by Breiman (Breiman, 2001). RF is a multifunctional machine learning method. It can perform the tasks of prediction and regression. In addition, RF is based on Bagging and it plays an important role in ensemble machine learning (Breiman, 2001; Lin et al., 2014; Svetnik et al., 2015). RF has been employed in several biomedicine research (Zhao et al., 2014; Liao et al., 2016).

RF generates many decision trees, which is very different from decision tree algorithm (Pal, 2005). When the RF is predicting a new object based on some attributes, each tree in RF will give its own classification result and ‘vote,’ and then the overall output of the forest will be the largest number of taxonomy. In the regression problem, the RF output is the average value of output of all decision trees (Liaw and Wiener, 2002; Svetnik et al., 2015).

#### Neural Network

**FIGURE 1:**
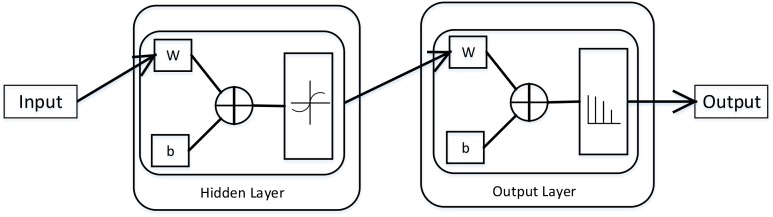
The structural of two–layer-feed-back network in MATLAB. This figure is from MATLAB, which can describe this network working principle preferably. Where, *W* is representation the weight and *b* is the bias variable.

Neural network is a math model, which imitates the animal’s neural network behaviors. This model depends on the complexity of the system to achieve the purpose of processing information by adjusting the relationship between the internal nodes (Mukai et al., 2012). According to the connections’ style, the neural network model can be divided into forward network and feedback network. In this paper, we used the Neural Pattern Recognition app in MATLAB, which is a two-layer-feed-back network with sigmoid hidden and softmax output neurons. The neural network structural is shown in (Figure 1).

In neural network, there are some important parts, namely input layer, hidden layer and output layer. The input layer is responsible for accepting input data. We can get the results from the output layer. The layer between the input layer and the output layer is called hidden layer. Because they are invisible to the outside. There is no connection between neurons on the same layer. In this network, the number of hidden layers set to 10, which can get a better performance. We suppose the input vector is $\overset{\to }{x}$, the weight vector is $\overset{\to }{w}$, and the activation function is a sigmoid function, then the output is:

$$y=sigmoid\left({\overset{\to }{w}}^{T}\cdot \overset{\to }{x}\right)$$

and the sigmoid is:

$$sigmoid(x)=\frac{1}{1+e^{-x}}$$

### Model Validation

In many studies, authors often used two validation methods, namely hold-out method and k-fold cross validation method, to evaluate the capability of the model (Kohavi, 1995; Bengio and Grandvalet, 2005; Kim, 2009; Chen et al., 2016; Refaeilzadeh et al., 2016; Yang et al., 2016, 2018; Su et al., 2018; Tang H. et al., 2018). According to the goal of each problem and the size of data, we can choose different methods to solve the problem. In hold-out method, the dataset is divided two parts, training set and test set. The training set is used to train the machine learning algorithm and the test set is used to evaluate the model (Kim, 2009). The training set is different from test set. In this study, we used this method to verity the universal applicability of the methods. In k-fold cross validation method, the whole dataset is used to train and test the classifier (Kim, 2009). First, the dataset is average divided into *k* sections, which called folds. In training process, the method uses the *k*-1 folds to training the model and onefold is used to test. This process will be repeat *k* times, and each fold has the chance to be the test set. The final result is the average of all the tests performance of all folds (Kohavi, 1995). The advantage of this method is the whole samples in the dataset are trained and tested, which can avoid the higher variance (Refaeilzadeh et al., 2016; Kavakiotis et al., 2017). In this study, we used the five-fold cross validation method.

### Feature Selection

Feature selection methods can reduce the number of attributes, which can avoid the redundant features. There are many feature selection methods. In this study, we used PCA and minimum redundancy maximum relevance (mRMR) to reduce the dimensionality.

#### Principal Component Analysis

PCA (Wang and Paliwal, 2003; Polat and Günes, 2007; You et al., 2018) obtains the *K* vectors and unit eigenvectors by solving the characteristic equation of the correlation matrix of the observed variables. The eigenvalues are sorted from large to small, representing the variance of the observed variables explained by *K* principal components, respectively (Smith, 2002).

The model for extracting principal component factors is:

$$F_{i}=T_{i1}X_{1}+T_{i2}X_{2}+T_{ik}X_{k}\left(i=1,2,...,m\right)$$

where, *F_i_* is the *i* principal component factor; *T_ij_* is the load of the *i* principal component factor on the *j* index; *m* is the number of principal component factors; *k* is the number of indicators.

The PCA method can reduce the original multiple indicators to one or more comprehensive indicators. This small number of comprehensive indicators can reflect the vast majority of the information reflected by the original indicators, and they are not related to each other, and they can avoid the repeated information (Jackson, 1993; Jolliffe, 1998). At the same time, the reduction of indicators facilitates further calculation, analysis and evaluation.

We used Statistical Product and Service Solutions (SPSS) to implement the PCA algorithm. SPSS is a general term for a series of software products and related services launched by IBM. It is mainly used for statistical analysis, data mining, predictive analysis and other tasks. SPSS has a friendly visual interface and is easy to operate.

#### Minimum Redundancy Maximum Relevance

mRMR (Jackson, 1993; Sakar et al., 2012; Li et al., 2016; Wang et al., 2018) ensures the features have the max Euclidean distances, or their pairwise have the minimized correlations. Minimum redundancy standards are usually supplemented by the largest relevant standards, such as maximum mutual information and target phenotypes. Two ways can achieve the benefits. First, with the same number of features, mRMR feature set can have a more representative target phenotype for better generalization. Secondly, we can use a smaller mRMR feature set to effectively cover the same space made by a larger regular feature set. For individual categorical variables, the similarity level between each feature is measured by using mutual information. Minimum redundancy is the choice to have the most different features. Similar to mRMR, researchers also developed Maximum Relevance Maximum Distance (MRMD) (Zou et al., 2016b) for features ranking. And they were employed in several biomedicine researches (Zou et al., 2016a; Jia et al., 2018; Tang W. et al., 2018; Wei et al., 2018).

### Measurement

In this study, we used sensitivity (SN), specificity (SP), accuracy (ACC), and Matthews correlation coefficient (MCC) to measure the classified effectiveness. And the formulas are as follow:

$$SN=\frac{TP}{TP+FN}$$

$$SP=\frac{TN}{TN+FP}$$

$$ACC=\frac{TN+TP}{TN+TP+FP+FN}$$

$$MCC=\frac{\left(TP\times TN\right)-\left(FN\times FP\right)}{\sqrt{\left(TP+FN)\times (TN+FP)\times (TP+FP)\times (TN+FN\right)}}$$

where true positive represents (TP) the number of identified positive samples in the positive set. True negative (TP) means the number of classification negative samples in the negative set. False positive (FP) is the number of the number of identified positive samples in the negative set. And false negative (FN) represents the number of identified negative samples in the positive set. It is often used to evaluate the quality of classification models. The accuracy is defined as the ratio of the number of samples correctly classified by the classifier to the total number of samples. In medical statistics, there are two basic characteristics, sensitivity (SN) and specificity (SP). Sensitivity is the true positive rate, and specificity is the true negative rate. The MCC is a correlation coefficient between the actual classification and the predicted classification. Its value range is [-1, 1]. When the MCC equals one, it indicates a perfect prediction for the subject. When the MCC value is 0, it indicates the predicted result is not as good as the result of random prediction, and -1 means that the predicted classification is completely inconsistent with the actual classification.

## Results and Discussion

In the tables, we used Luzhou to represent the dataset from hospital physical examination data in Luzhou, China and Pima Indians represents the Pima Indians diabetics data. The two datasets contain 14 and 8 attributes, respectively.

For better comparison, firstly, we used all features for predicting diabetes. And the results are shown in Table 1.

**Table 1:** Predict the diabetes by using all features.

Dataset	Classifier	ACC	SN	SP	MCC
Luzhou	RF	0.8084	0.8495	0.7673	0.6189
	J48	0.7853	0.8153	0.7563	0.5726
	Neural network	0.7841	0.8231	0.7451	0.5699
Pima Indians	RF	0.7604	0.7578	0.7631	0.5210
	J48	0.7275	0.7027	0.7523	0.4569
	Neural network	0.7667	0.7828	0.7508	0.5349


**FIGURE 2:**
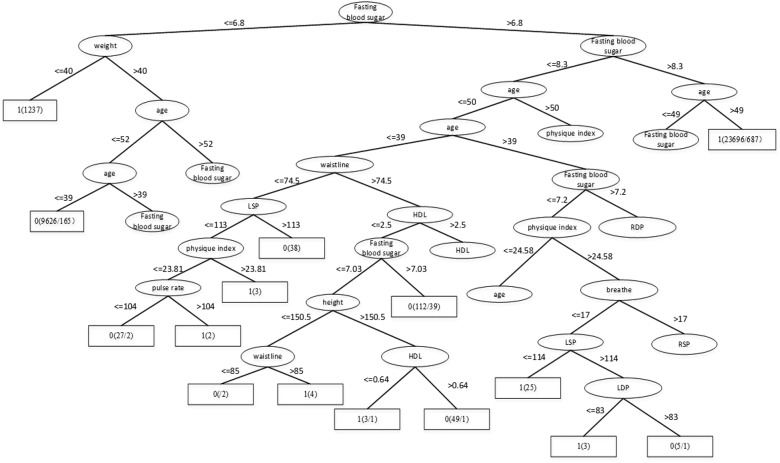
Decision tree structure by using all features and Luzhou dataset. In this figure, we can find the fasting blood sugar is an important index for predicting diabetes And weight, age also have the higher information gain and play vital roles in this method.

**FIGURE 3:**
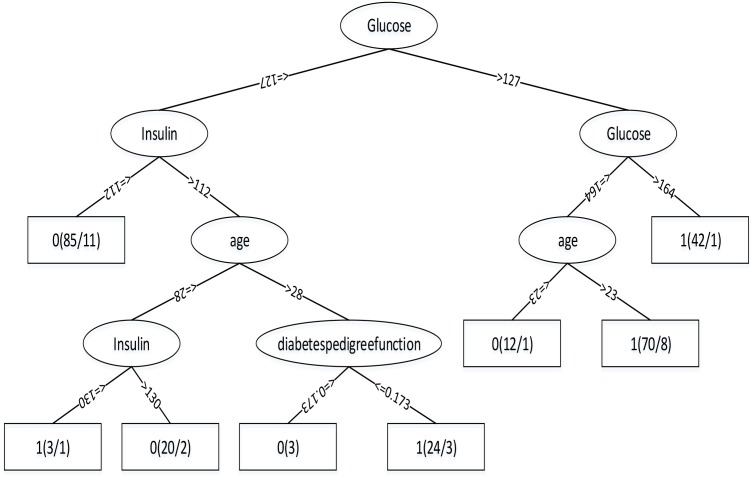
Decision tree structure by using all features and Pima Indians dataset. From this figure, we can find in this method glucose as the root node, which can indicate the index has the highest information gain and insulin and age play important roles in this method.

Through the Table 1, we can get better results. In addition, RF has the best result among the three classifiers when the dataset is Luzhou physical examination. When the dataset is Pima Indians, random forest has similar effects to neural networks. And the decision tree structure of Luzhou dataset is shown in Figure 2, the decision tree structure of Pima Indians dataset is shown in Figure 3. According to Figures 2, 3, we can find the root node is glucose, which can show the glucose has the max information gain, so it confirm the common sense and the clinical diagnosis basis. But there are diabetic patients whose fasting blood glucose is less than 6.8 in Luzhou dataset, we considered the reason maybe they injected insulin before the physical examination to control blood sugar levels.

According to consulting relevant information, we know there are three indicators to determination the diabetes mellitus, which are fasting blood glucose, random blood glucose and blood glucose tolerance. Because the data only has fasting blood glucose in Luzhou dataset and the Pima Indians dataset only has blood glucose tolerance, we used fasting blood glucose and blood glucose tolerance to prediction, respectively. And the results are shown in Table 2.

**Table 2:** Predict the diabetes by using blood glucose.

Dataset	Classifier	ACC	SN	SP	MCC
Luzhou	RF	0.7597	0.8795	0.6400	0.5350
	J48	0.7610	0.8818	0.6401	0.5379
	Neural network	0.7572	0.8870	0.6274	0.5327
Pima Indians	RF	0.6728	0.6765	0.6692	0.3461
	J48	0.6895	0.7320	0.6355	0.3733
	Neural network	0.7198	0.6950	0.7446	0.4411


According to the Table 2, we found in Luzhou dataset J48 has a better performance than the others do, and the accuracy is above 0.76. In the Pima Indians dataset, only using blood glucose tolerance is not good.

Then, we used mRMR to select features. We get the score of each feature. According to the matrix, we chose the first five features, which are height, HDL, fasting glucose, breathe, and LDL, to predict diabetes using Luzhou dataset and select the first three attributes, which are glucose, 2-h serum insulin and age, to predict the Pima Indians dataset. The results are shown in Table 3.

**Table 3:** Predict diabetes of using mRMR to reduce dimensionality.

Dataset	Classifier	ACC	SN	SP	MCC
Luzhou	RF	0.7508	0.8334	0.6681	0.5085
	J48	0.7613	0.8795	0.6431	0.5379
	Neural network	0.7570	0.8828	0.6313	0.5312
Pima Indians	RF	0.7721	0.7458	0.7985	0.5451
	J48	0.7534	0.7228	0.7846	0.5095
	Neural network	0.7390	0.8073	0.6708	0.4837


When we use the Luzhou dataset, J48 has the best performance. But the results are not better than using all features. In the Pima Indians dataset, this method, which used RF as the classifier, has the best performance.

Then we used PCA to reduce the features. Because height and weight are related to physical index, we did not use height and weight to using PCA in Luzhou dataset. We used SPSS to analyzing the factors. According to the KMO and Bartlett test, the two datasets can use PCA to reduce the features. And we can get the composition matrix and eigenvalues. According to the composition matrix and total variance interpretation, we can get the new five features for Luzhou dataset and three features for Pima Indians dataset. We use the new features to conduct experiment, and the results are shown in Table 4.

**Table 4:** Predict diabetes of using PCA to reduce dimensionality.

Dataset	Classifier	ACC	SN	SP	MCC
Luzhou	RF	0.7395	0.7435	0.7354	0.4790
	J48	0.7388	0.7335	0.7441	0.4777
	Neural network	0.7414	0.7370	0.7457	0.4828
Pima Indians	RF	0.7144	0.7057	0.7231	0.4291
	J48	0.7167	0.7381	0.6954	0.4353
	Neural network	0.7475	0.7381	0.7569	0.4968


The ACC of Luzhou dataset is less than the above methods. The results show PCA is not suitable for this data. For Pima Indians dataset, the accuracy is better than only use glucose. In this second, neural network has the best performance for predicting diabetes.

In order to explore the importance of other indexes in predicting diabetes, we designed the following experiments by using Luzhou dataset. Firstly, we used the all features without blood glucose to predict diabetes, and the results are shown in Table 5.

**Table 5:** Predict diabetes of using all features without blood glucose.

Dataset	Classifier	ACC	SN	SP	MCC
Luzhou	RF	0.7225	0.7228	0.7222	0.4450
	J48	0.6917	0.6880	0.6953	0.3834
	Neural network	0.6986	0.6646	0.7326	0.3981


And then, we deleted the blood glucose, LDL and HDL which need to go to the hospital for testing data. So there are 11 features in this experiment, and the results are shown in Table 6.

**Table 6:** Predict diabetes of using 11 features.

Dataset	Classifier	ACC	SN	SP	MCC
Luzhou	RF	0.7104	0.7082	0.7125	0.4207
	J48	0.6916	0.6880	0.6953	0.3833
	Neural network	0.6983	0.6685	0.7281	0.3973


According to the Tables 5, 6, we found the RF is able to predict better diabetes. Although the accuracy is not the best, we can use the prediction as a reference.

**FIGURE 4:**
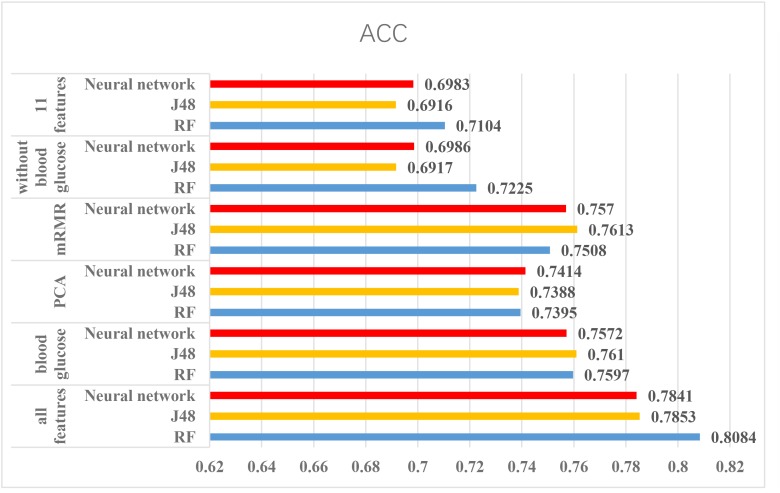
The results of using Luzhou dataset. According to this figure, we found the method, which used all features and random forest has the greatest performance. And the methods without blood glucose are not good.

**FIGURE 5:**
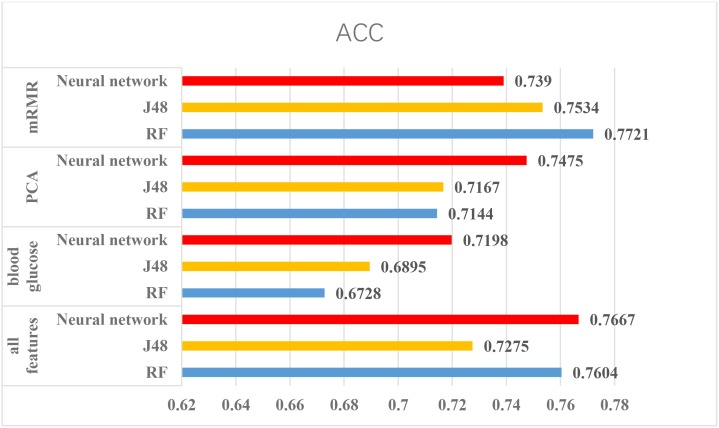
The results of using Pima Indians dataset. From the figure, mRMR is friendly for this dataset and method only using glucose is not suitable for this dataset.

According to the above experiments, we summarized the above results and get Figures 4, 5, which can more clearly demonstrate the accuracy of each method in order to make a better comparison.

From the Figures 4, 5, we can find PCA is not very suitable to the two dataset. And using all features has a good performance, especially for the Luzhou dataset. There is not much difference among random forest, decision tree and neural network when the feature set contains blood glucose. When we used the features without blood glucose, random forest has the best performance. But relatively speaking, the neural network performs poorly.

According to the Figure 4, we selected several methods that performed better and conducted independent testing experiments by using Luzhou dataset. So we chose three methods (all features, mRMR and blood glucose) to conduct independent test experiments. The results are shown in Table 7.

**Table 7:** Predict diabetes of using independence test data.

Method	Classifier	ACC	SN	SP	MCC
mRMR	RF	0.8857	0.9568	0.8146	0.7794
	J48	0.7547	0.8647	0.6447	0.5223
	Neural network	0.7470	0.8655	0.6284	0.5085
All features	RF	0.8963	0.9226	0.8700	0.7937
	J48	0.8011	0.8135	0.7887	0.6025
	Neural network	0.7725	0.7942	0.7508	0.5455
Blood glucose	RF	0.7537	0.8704	0.6371	0.5218
	J48	0.7535	0.8713	0.6358	0.5218
	Neural network	0.5010	0.9388	0.0631	0.0040


According to Table 7, we found the method using all features still has a better result. And the method only using blood glucose is not good, especially using neural network as classifier. The reason for this result may be that the blood glucose contains too little information.

Because Luzhou dataset is collected by ourselves, it is unable to use this data for comparison experiments. In order to compare with the methods in other papers, we used Pima Indians dataset for 10-fold cross validation experiments. The results are shown in Table 8.

**Table 8:** Predict diabetes of using all features without blood glucose.

Method	ACC	Reference
mRMR (RF)	0.7852	Our study
mRMR (J48)	0.7806	Our study
All feature (RF)	0.7604	Our study
All feature (J48)	0.7275	Our study
AWAIS(10xCV)	0.7587	Polat and Kodaz, 2005
NNEE	0.7557	Jiang and Zhou, 2004
AIRS(13xCV)	0.7410	Watkins and Boggess, 2002


## Conclusion

Diabetes mellitus is a disease, which can cause many complications. How to exactly predict and diagnose this disease by using machine learning is worthy studying. According to the all above experiments, we found the accuracy of using PCA is not good, and the results of using the all features and using mRMR have better results. The result, which only used fasting glucose, has a better performance especially in Luzhou dataset. It means that the fasting glucose is the most important index for predict, but only using fasting glucose cannot achieve the best result, so if want to predict accurately, we need more indexes. In addition, by comparing the results of three classifications, we can find there is not much difference among random forest, decision tree and neural network, but random forests are obviously better than the another classifiers in some methods. The best result for Luzhou dataset is 0.8084, and the best performance for Pima Indians is 0.7721, which can indicate machine learning can be used for prediction diabetes, but finding suitable attributes, classifier and data mining method are very important. Due to the data, we cannot predict the type of diabetes, so in future we aim to predicting type of diabetes and exploring the proportion of each indicator, which may improve the accuracy of predicting diabetes. We uploaded the Pima Indians dataset in http://121.42.167.206/PIMAINDIANS/data.html.

## Author Contributions

QZ designed the experiments. KQ and YL performed the experiments. KQ wrote the paper. DY and YJ analyzed the data. HT provided the data.

## Conflict of Interest Statement

The authors declare that the research was conducted in the absence of any commercial or financial relationships that could be construed as a potential conflict of interest.
